# In Vitro HIV-1 Selective Integration into the Target Sequence and decoy-effect of the modified sequence

**DOI:** 10.1186/1742-4690-8-S2-P85

**Published:** 2011-10-03

**Authors:** Tatsuaki Tsuruyama

**Affiliations:** 1 Department of Pathology, Graduate School of Medicine, Kyoto University, Kyoto city, Kyoto Prefecture, Japan

## Background

There have been a few reports that the HIV-1 genome can be selectively integrated into the genomic DNA of host cell. However, actual target sequence of integration has not been reported. The target sequence within the second intron of *Stat5a* gene of MLV integration has been already reported by us [1,2](Patent No. 4631084, 2010, Japan, W02006/022249).

## Materials and methods

On basis of the CA-rich sequence motif that was observed in MLV integration target sequence, we prepared a substrate repeat sequence DNA for *in vitro* HIV-1 integration, 5’-(GTCCCTTCC*CAGT*)6(ACTGGGAAGGGAC)6-3’ and a set of modified sequence DNAs by deletion of *CAGT* in the repeat unit. This *CAGT* and ACTG (shown in italics in the above sequence) in the repeat units originated from the HIV-1 proviral genome ends. We devised *in vitro* integration by using these sequence DNAs, HIV-1 provirus DNA, and recombinant HIV-1 integrase.

## Results

*In vitro* integration occurred at the target sequence DNA at significant higher frequency and selectivity in comparison to random-sequence DNAs. Although the target sequence consisted of repeat segments, *in vitro* integration selectively occurred in the middle segment of the repeat sequence. On the other hand, both frequency and selectivity decreased markedly when using sequences with deletion of *CAGT* in the middle segment of the target sequence. Moreover, on incubation with the *CAGT*-deleted DNAs and target sequence, the integration efficiency and selectivity for the target sequence were significantly reduced. This interesting data indicated interference effects by the mixed sequence *CAGT*-deleted DNAs. Besides, efficiency and selectivity of integration into the target repeat sequence was found to vary discontinuously with changes in manganese dichloride concentration in the reaction buffer for *in vitro* integration. Because the structure transition at the critical concentration was exclusively observed in the target sequence DNA by electrophoresis, these discontinuous changes in *in vitro* integration were probably due to *fluctuation* in the secondary structure of the target DNA segment. Such structural isomers may be favorable for selective integration into the target sequence DNA.

## Conclusions

There is a considerable selectivity in *in vitro* HIV-integration into the specified sequence. Similar DNA sequences can interfere with the process of selective integration. Dependency of *in vitro* integration upon the secondary structure of the target DNA is one of the models of *in vivo* integration that is promoted by open chromatin structure that is induced by transcriptional factor bound to the neighboring DNA segment. In addition, the present *in vitro* integration system can be useful for monitoring the integration activity or test of integrase inhibitor [3].
